# Label‐Free Identification of White Blood Cells Using Machine Learning

**DOI:** 10.1002/cyto.a.23794

**Published:** 2019-05-13

**Authors:** Mariam Nassar, Minh Doan, Andrew Filby, Olaf Wolkenhauer, Darin K. Fogg, Justyna Piasecka, Catherine A. Thornton, Anne E. Carpenter, Huw D. Summers, Paul Rees, Holger Hennig

**Affiliations:** ^1^ Department of Systems Biology & Bioinformatics University of Rostock 18051 Rostock Germany; ^2^ Imaging Platform at the Broad Institute of Harvard and MIT 415 Main St, Cambridge Massachusetts 02142; ^3^ Flow Cytometry Core Facility, Faculty of Medical Sciences Newcastle University Newcastle upon Tyne NE1 7RU UK; ^4^ Stellenbosch Institute for Advanced Study (STIAS) Stellenbosch South Africa; ^5^ Autograph Biosciences, Inc. Montreal Quebec Canada; ^6^ Centre for Nanohealth, Swansea University Singleton Park, Swansea SA2 8PP UK

**Keywords:** high‐content analysis, machine learning, imaging flow cytometry, white blood cells, white blood cell count, personalized medicine, label‐free classification, lymphocytes, liquid biopsy

## Abstract

White blood cell (WBC) differential counting is an established clinical routine to assess patient immune system status. Fluorescent markers and a flow cytometer are required for the current state‐of‐the‐art method for determining WBC differential counts. However, this process requires several sample preparation steps and may adversely disturb the cells. We present a novel label‐free approach using an imaging flow cytometer and machine learning algorithms, where live, unstained WBCs were classified. It achieved an average F1‐score of 97% and two subtypes of WBCs, B and T lymphocytes, were distinguished from each other with an average F1‐score of 78%, a task previously considered impossible for unlabeled samples. We provide an open‐source workflow to carry out the procedure. We validated the WBC analysis with unstained samples from 85 donors. The presented method enables robust and highly accurate identification of WBCs, minimizing the disturbance to the cells and leaving marker channels free to answer other biological questions. It also opens the door to employing machine learning for liquid biopsy, here, using the rich information in cell morphology for a wide range of diagnostics of primary blood. © 2019 The Authors. *Cytometry Part A* published by Wiley Periodicals, Inc. on behalf of International Society for Advancement of Cytometry.

White blood cell (WBC) differential count is a clinical test that measures the number and percentage of each WBC type in a person's sample of blood. WBCs play an important role in the body's immune system and in the defense against infections. They are categorized into five main types: lymphocytes (including B and T cells), eosinophils, neutrophils, monocytes, and basophils. These WBC populations have characteristic concentration ranges in healthy persons; deviations, whether high or low, are clinically significant 1. The reference range for WBC differential count is as follows: lymphocytes (20–40%), eosinophils (1–6%), monocytes (2–10%), and neutrophils (40–80%) (https://emedicine.medscape.com/article/2085133-overview).

Conventionally, WBC differential count often involves flow cytometry using fluorescently tagged antibodies that are known to differentially label the WBC populations. This procedure is widely adopted in the clinical routine with well‐established standard laboratory protocols. Traditional flow cytometers classify stained cells in a high‐throughput, low‐content manner based on a small number of light‐scatter properties (forward scatter and side scatter), and fluorescence intensity features. Modern cytometers, such as mass cytometers, can measure up to 40 parameters in a single panel 2; however, similar to fluorescence‐based cytometers, they are limited to analysis of cell phenotype based on expression levels of antibody‐labeled molecules.

Imaging flow cytometry combines the sensitivity and high‐content morphology of digital microscopy with the high‐throughput and statistical power of the conventional flow cytometer. Imaging flow cytometers can simultaneously acquire up to 12 images of each single cell that passes through the instrument. The first commercially available imaging flow cytometers were built about a decade ago. Yet, these instruments are mostly used in a research context rather than in clinical practice.

Although imaging flow cytometers are capable of capturing information‐rich cell images, common analytic pipelines are rarely seen exploiting its high‐content potential. Better approaches have been proposed, where data‐driven feature extraction, feature selection, and machine learning have been applied to unbiasedly identify morphological profiles that are subtle and less visible to human observation 3, 4, 5.

Previous work to develop a label‐free WBC differential count achieved 99% classification accuracy distinguishing lymphocytes, granulocytes, and monocytes 6. However, this approach requires commercially unavailable instrumentation and uses shuffle and split to evaluate the prediction model. Shuffle and split means that all information is shuffled and then split into training and validation set. This method is prone to overfitting as the classifier has already seen blood cells from a particular subject in the training set and then predicts on the same subject 7. Overfitting leads to high accuracy in the prediction, however, the trained model then fails in a realistic setting where the patient is not previously known to the classifier.

Here, we present a novel machine learning analysis pipeline for label‐free WBC differential counts, in which overfitting is controlled by subject‐wise cross‐validation procedure. Using this pipeline, unstained WBCs assayed by an imaging flow cytometer could be classified into the unique subtypes with high F1‐score. Interestingly, we were able to distinguish B and T lymphocytes (although with a lower F1‐score as compared to the other white blood cell types), a task previously considered impossible for unlabeled samples 8, 9. Our approach was validated with stained blood samples from 13 and unstained samples from 85 healthy volunteers generated in a clinical study at Swansea University (United Kingdom).

We also provide an open‐source workflow to address the technical barrier to promote broader adoption of machine learning‐based blood cell identification. A user‐friendly workflow represents an important step toward translation into clinical practice and toward extension of the approach for liquid biopsy to identify tumor products such as circulating tumor cells in the blood 10.

## materials and methods

### Data Acquisition and Preprocessing Raw Images in IDEAS

Blood samples from more than 100 healthy blood donors were collected, and all images were anonymized following standard procedures by assigning them ID numbers. The ethical approval for this study has been granted by the ethics committee of the University of Rostock (Germany; approval number: A 2017–0028) and Swansea University (United Kingdom).

Blood was drawn from healthy donors and collected directly into heparinized tubes. The sample was then split across multiple tubes (500 ul/tube) and each stained individually with optimized concentrations of Fluorescein isothiocyanate (FITC)‐labeled antibodies against one of the following cell surface markers: CD3 (T cells), CD14 (monocytes), CD15 (neutrophils), and CD19 (B cells). One tube per sample was left unstained. After 20 min of incubation in the dark at room temperature (RT), red blood cell lysis was performed using BD Lyse‐fix solution as per the manufacturer's instructions. Briefly, the 10× lysis buffer stock was diluted 1:10 in reagent grade water to a 1× working concentration. The whole blood was then incubated at a ratio of one part blood to nine parts lysis buffer for 10 min at RT. Samples were then spun down at 350 g for 5 min and washed a further 2 times with PBS +2% FBS before a final resuspension at 5 × 106/ml. Cell suspensions were then loaded into an Amnis ImageStream100 (Luminex Corporation, Austin TX) imaging flow cytometer, which can acquire up to six multi‐spectral images (channels) for every cell 11 at a rate of up to 100 cells per second. The images were saved as raw image files (.RIF), which were then opened and processed in the IDEAS software (accompanying the ImageStream) in order to perform spectral compensation using a FITC only stained control sample that had been collected with both bright‐field and scatter sources turned off.

The features used in this work were derived from the brightfield (transmitted light) image, and the darkfield (also known as side‐scatter [SSC]) image; in which the light source is positioned orthogonally to the detection camera). For the ground‐truth determination, FITC‐labeled cell surface markers were detected in one of the four fluorescence channels on the camera.

### IDEAS Focused Single Cell Gating

Multispectral images acquired using the ImageStream100 were subsequently analyzed in the IDEAS software version 6.2.65.0 program. Gradient root mean squared (RMS) on the BF image measures the sharpness of focus by calculating the average gradient of pixel intensities across the image. Poorly focused images are blurry and therefore have relatively low gradient. We used a threshold on the brightfield image of 55 arbitrary units to exclude cells of poor focus quality. Images of single cells were separated from cellular debris, and cell doublets or aggregates using the aspect ratio and area measurements of the object masks for the brightfield image, and were used for downstream analysis.

**Figure 1:**
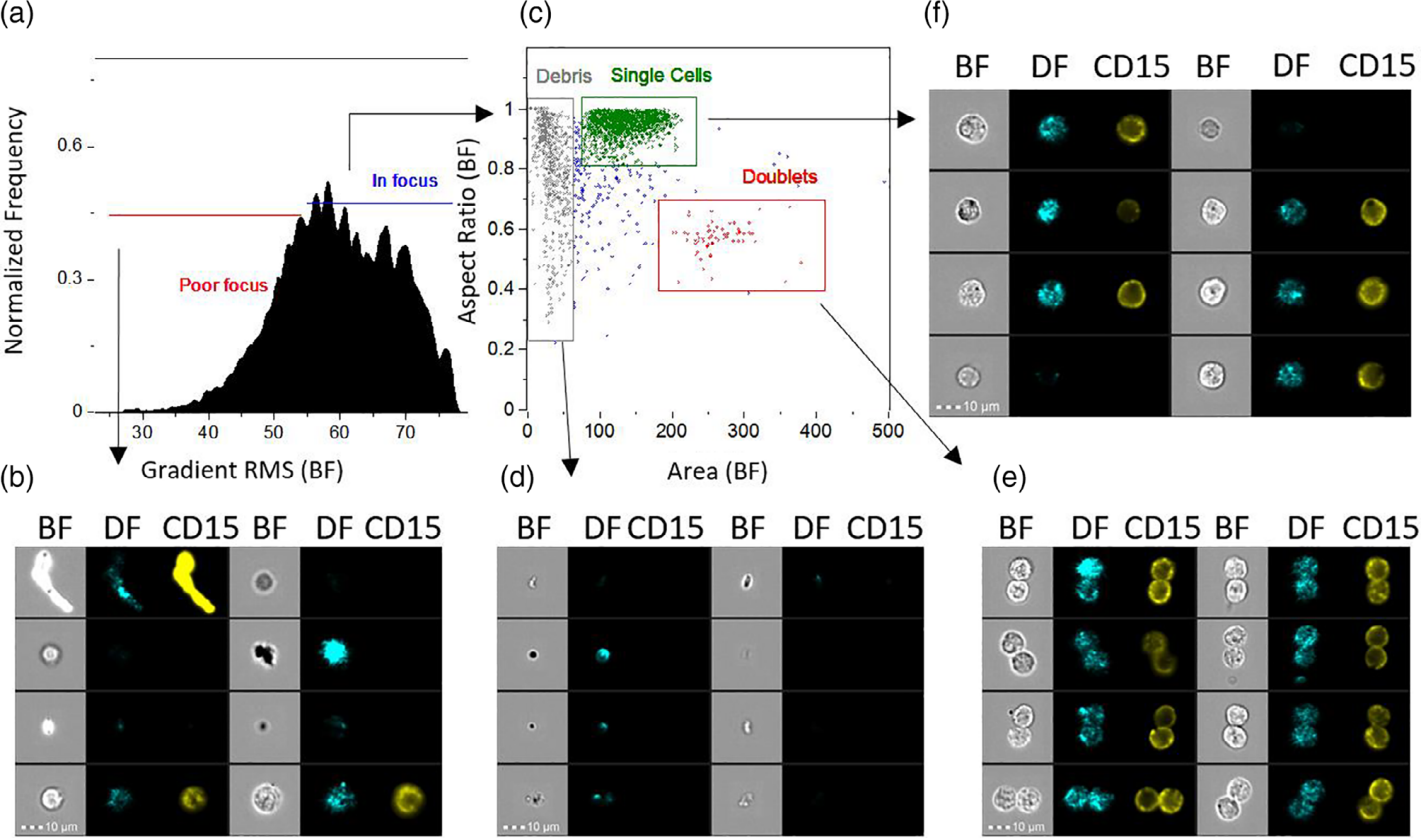
Workflow for selecting cell images for machine learning. Images captured by imaging flow cytometry were curated by human experts to remove out‐of‐focus events and artifacts (**a**), (**b**). In‐focus events (**c**) were then analyzed for exclusion of debris (**d**), and doublets or coincident events (**e**). Images of single cells in sharp focus (**f**) were then saved as .CIF files for later inputs of CellProfiler. Abbreviations: BF = Brightfield; DF = Darkfield. The fluorescence channel shown is CD15 in this example. Similar gating was used for other surface marker stains. Scale bar is 10 μm).

In addition, we devised the so‐called templates, which contain quality control steps and the gates. A gate is a user‐defined region within a histogram or bivariate dotplot, which contains a cell subpopulation defined using a small number of characteristic features. These templates were used to build the training set of WBC subpopulations based on the fluorescently stained biomarkers included for ground truth purposes. All the extracted cell populations were exported to separate files in the compensated image files (.CIF) format. The preprocessing workflow performed in IDEAS is shown in Figure 1 in addition to sample images.

The training set is the result of data preprocessing we performed and contains the white blood cells from 13 donors, all with both unstained and blood samples parts stained against CD3, CD14, CD15, and CD19. The number of cells is shown in the Supporting Information Table SF.1.

### Stitching–Making Montages out of Single‐Cell Images

This step of the workflow consisted of extracting single images from each .CIF file—the ImageStream proprietary image‐container format. We developed a Python script, *Stitching* (available at https://github.com/CellProfiler/stitching), to read .CIF files and generate montages in .TIF format, each is a collection of 900 tiled cell images; one montage per channel.

An additional bash script (see Supporting Information) was made to loop over the .CIF files, apply stitching and generate the corresponding montages for each clinical sample.

### Feature Extraction from each Single Cell Using CellProfiler

In this step of the workflow, the WBC montages (.TIF files) were imported into CellProfiler 12, which is an open‐source software designed for high‐throughput cell image analysis using the concept of a pipeline. CellProfiler consists of several image‐processing modules, each processes the cell image in a specified manner and measures specific morphological parameters.

We developed a CellProfiler pipeline, available at http://cellprofiler.org/imagingflowcytometry/index.html and included in the Supporting Information, to measure 213 morphological features across the categories shape, size, intensity, and texture per WBC per channel. The pipeline analyzed image montages, identified cellular objects, filtered bad quality cells and generated tables of cell features as .csv files.

### Application of Machine Learning to Classify WBCs

In this step of the workflow, several machine‐learning algorithms were applied on the feature data previously generated to develop and evaluate WBC classification models. Python programming environment and its scikit‐learn library 13 were utilized to form a series of diverse machine learning frameworks.

Overfitting generally happens when the machine‐learning model learns from the noise in the data and leads to poor generalization on unseen data. Cross‐validation is an evaluation strategy to test the model against overfitting (in methods). We thus applied subject‐wise cross validation to evaluate and compare six machine‐learning algorithms: AdaBoost, Gradient Boosting (GB), K‐Nearest Neighbors (KNN), Random Forest (RF), and Support Vector Machine (SVM).

The data set was highly imbalanced with typically (40–80%) cells in the most prominent class and only (1–6%) cells in the least prominent class per subject due to the normal ranges of WBC classes in the human blood. To solve this issue, we balanced the WBC classes using random undersampling to investigate the influence of imbalance on our data set. We run undersampling 10 times for robust results, for each run, we determined the best classifiers. After that, we applied majority voting to determine the overall best classifier.

To evaluate the performance of machine learning models, we applied subject‐wise cross‐validation. Subject‐wise cross validation was performed in 13 iterations (the training data set contains 13 blood donors). In each iteration, the data set from one blood donor was separated for validation, and the machine‐learning classifier is trained on the others; (illustrated in Supporting Information Fig. SD.1). For each iteration, we generated the F1‐score and built the average over all iterations; this average F1‐score—combining the precision and recall metrics in one value—was then used as an evaluation metric, which was suitable for both balanced and imbalanced classes.

We compared the top classifier for imbalanced classes with the top classifier for balanced classes to yield the best‐overall machine‐learning classifier.

We carried out this procedure as a two‐stage classification: first, classifying the WBCs into eosinophils, lymphocytes, monocytes, and neutrophils; and second, classifying the identified lymphocytes into B and T cells. In the latter case, we restricted the training and evaluation only to cells labeled as lymphocytes.

### Dissemination

All scripts are freely available on GitHub (https://github.com/mariamnassar/imagingFlowCytometry/tree/master/machineLearning/WhiteBloodCells) and as Supporting Information files.

## Results

**Figure 2:**
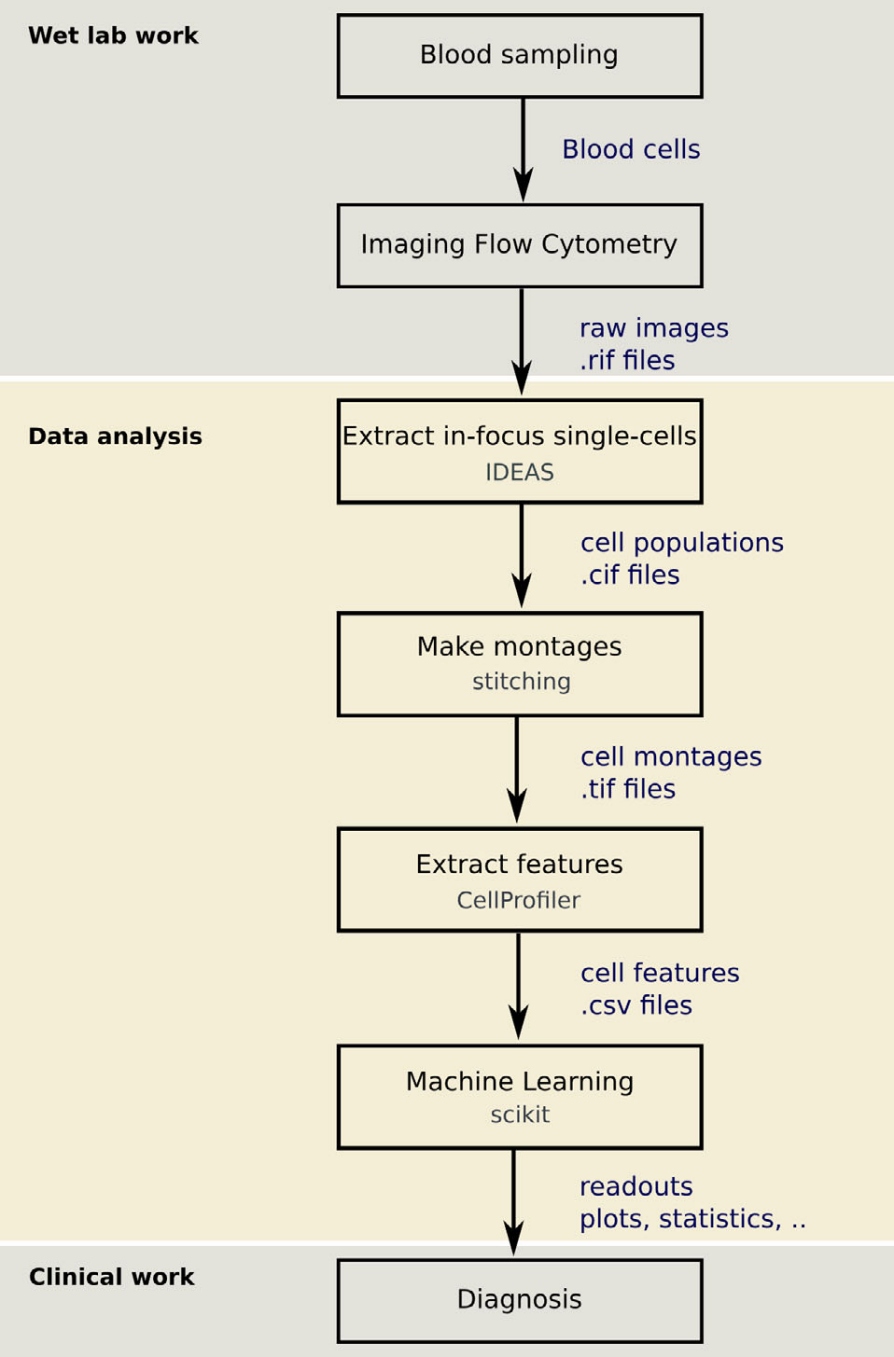
The steps of the developed workflow combining imaging flow cytometry and machine learning to classify WBCs.

Using data from a clinical study at Swansea University (United Kingdom), we developed a workflow to compute WBC differential counts from blood samples (Fig. 2).

We applied subject‐wise cross‐validation, where the correct WBC class labels for each cell were determined by staining the WBCs and gating them based on the emitted fluorescence. Of the 100 blood donor data sets, only 13 have stained WBCs of all types, which were used for cross validation.

**Figure 3:**
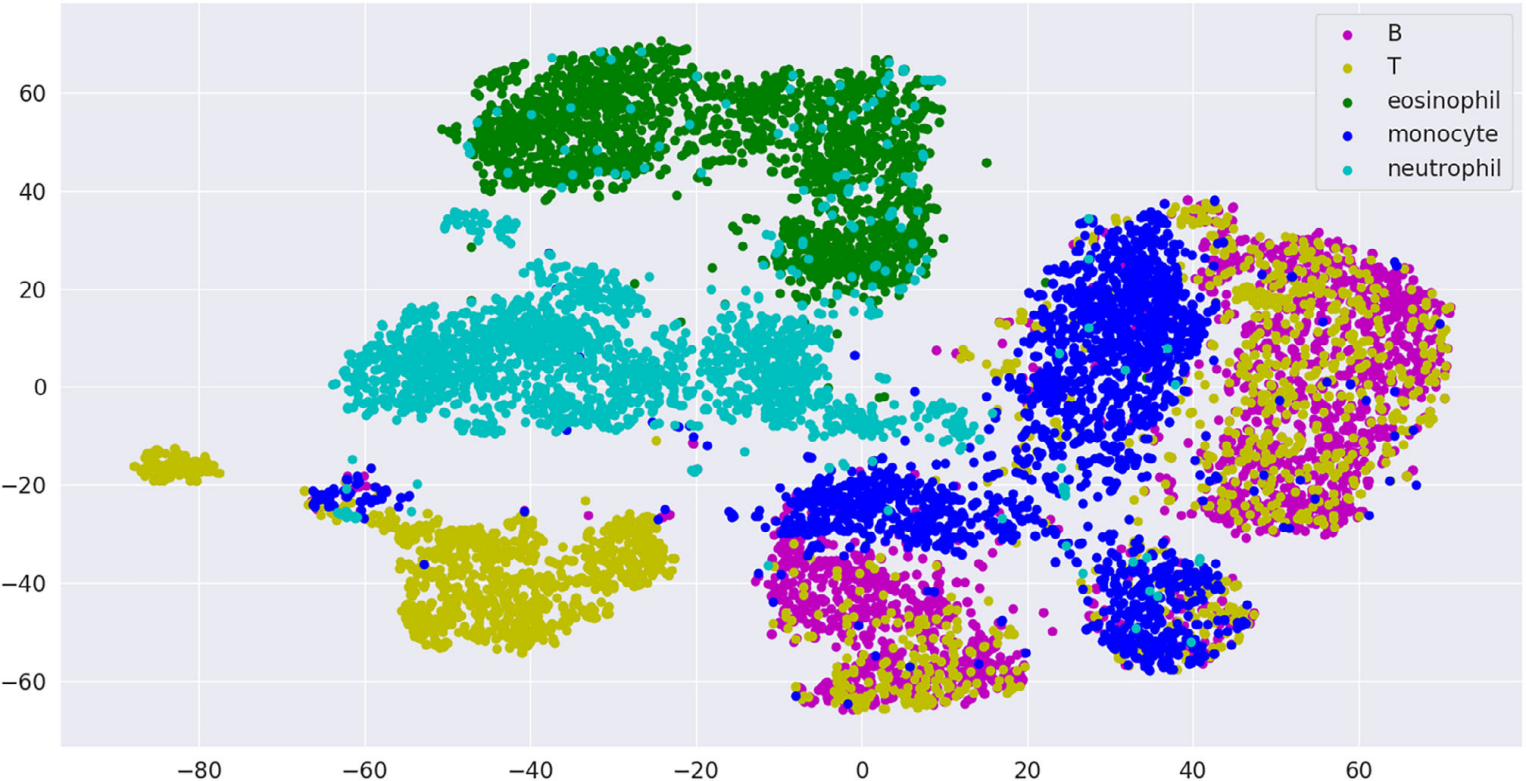
T‐SNE visualization of the training data set.

We visualized the normalized training data set using t‐distributed Stochastic Neighbor Embedding (t‐SNE) 14. The t‐SNE plot is shown in Figure 3, where we observe one eosinophil cluster, one neutrophil cluster, three almost separated monocyte clusters, and lymphocytes, which are separated into two mixed B and T clusters in addition to two T clusters. The interpretation of the cluster is subject to further research. An annotated t‐SNE plot with cell images visualization is available at http://projector.tensorflow.org/?config=https://raw.githubusercontent.com/mariamnassar/imagingFlowCytometry/master/tensorboard_projector/embedding_config.json

The best algorithm among those tested achieved an F1‐score of about 97% (Tables D.1 and D.2, and see Materials & Methods, and limitations in the Discussion section) for classifying WBC into main types (eosinophils, lymphocytes, monocytes, and neutrophils). This model was trained using Gradient Boosting combined with random undersampling of the WBC classes.

For classifying lymphocytes into B and T cells, Gradient Boosting combined with random undersampling performed best with an F1‐score of about 78% (Tables D.3 and D.4).

### Feature Selection

Feature selection provides an insight into which features are important for classification model. Our classical machine‐learning approach with hand‐crafted features has the transparency advantage, that is, it is clearly interpretable how and why a decision is made by the model, and whether the decision is robust and under which conditions it could possibly break.

In order to better understand how these models were established and function, we extracted the feature importance scores using Gradient Boosting and generated the union of the most important 10 features over all random undersampling test runs, sorted by their frequencies (Tables 1 and 2). We observed that intensity features of both brightfield and darkfield channels were particularly important for the classification of WBC main types in addition to the granularity of the cell center (Granularity 1). For lymphocyte T versus B cell classification, the overall shape of the cell was important in addition to mostly brightfield intensity and granularity features. This was indeed in accordance with previous findings: B and T cells have differences in amount of cytoplasm, nuclear size, homogeneity, nuclear folds, nuclear membrane, or presence and uniformity of nucleoli 9. Furthermore, B and T cells generally have been noticed to possess different textures (contrast, correlation, energy) and refractive indexes 15, especially when they are activated and specialized into subtypes at different immunological checkpoints.

**Table 1:** Highest ranked morphological features for WBC classification. The table shows the most important features used by gradient boosting for the WBC main types classification using random undersampling. Detailed explanation of the features can be found in the CellProfiler user manual available at http://cellprofiler.org/manuals/

Feature	Channel
MAD intensity	Darkfield
Std intensity	Darkfield
Integrated intensity	Darkfield
Lower quartile intensity	Brightfield
Granularity 1	Darkfield
Mean Intensity	Brightfield
Upper quartile intensity	Darkfield
Granularity 1	Brightfield
Std intensity edge	Brightfield
Integrated intensity edge	Darkfield

The first column contains the feature names and the second column contains the associated channel. Features were measured in the entire cell (no subcompartments of cells were defined). The features were sorted by their frequencies in 10 random undersampling test runs.

**Table 2:** Highest ranked morphological features for lymphocyte classification. The table shows the most important features used by gradient boosting for lymphocyte classification using random undersampling

Feature	Channel
Std intensity edge	Brightfield
Lower quartile intensity	Brightfield
MeanFrac Radial Distribution 4of4	Brightfield
Mean intensity	Brightfield
Integrated intensity edge	Darkfield
Granularity 1	Brightfield
FracAtD Radial Distribution 4of4	Brightfield
Granularity 1	Darkfield
DifferenceVariance Texture 3_0	Brightfield
Granularity 3	Brightfield

The first column contains the feature names and the second column contains the associated channel. Features were measured in the entire cell (no subcompartments of cells were defined). The features were sorted by their frequencies in 10 random undersampling test runs.

### Validation on Unstained Data

**Figure 4:**
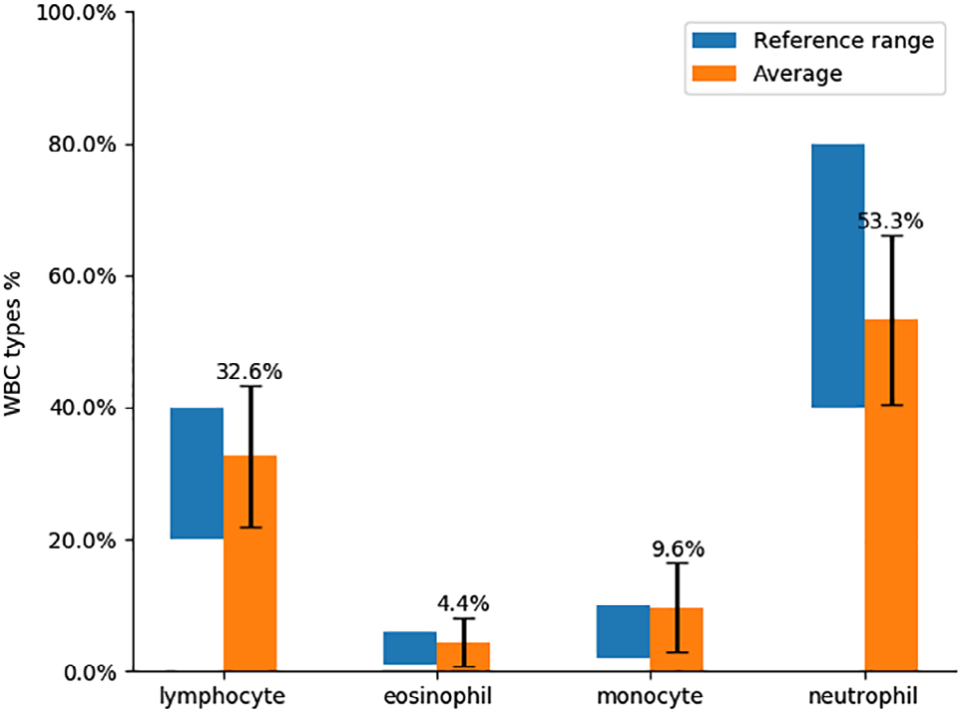
Average WBC count over 85 unstained blood donors compared with the average WBC count range.

We validated our WBC analysis on blood samples from 85 healthy blood donors. We applied the presented workflow to extract features from the acquired images. Then we used the Gradient Boosting classifier trained on the features from 13 healthy blood donors to classify the WBCs from 85 blood donors and generated the WBC count for each one. After that, we computed the average WBC count and compared it with the reference WBC count in Figure 4. We observe that, for every WBC type, the average count corresponds to the reference ranges.

## discussion and outlook

In this work, we presented a workflow that combines imaging flow cytometry with machine learning to analyze, classify, and generate WBC differential counts to be used by medical doctors for disease diagnosis and monitoring. We compared six machine‐learning classifiers and showed that the best among them, GB combined with random undersampling, can classify WBCs into the main types, yielding 97% average F1‐score. In addition, we showed that Gradient Boosting combined with random undersampling is able to classify lymphocytes with an average cross validation F1‐score of 78%, demonstrating for the first time that lymphocytes can be morphologically distinguished.

The presented workflow improves the state‐of‐the‐art flow cytometry method by classifying cells without using fluorescent markers. This might help to reduce mechanical disturbances to the cells and make the sample preparation procedure faster and more robust.

A natural next step for future work is to apply deep learning methods with the aim to increase the performance of the model, particularly given that we release the data publicly (https://github.com/mariamnassar/imagingFlowCytometry). Although classical image segmentation and machine learning have advantages in terms of the ability to understand the features used in making classification decisions about each cell, there is considerable ongoing work in deep learning on methods of interpretability and bias detection. Examples include FairML 16 Google's What‐If tool for Tensorflow (https://pair-code.github.io/what-if-tool/) and IBM's AIF360 tool (http://aif360.mybluemix.net/). Bias detection also helps to assess the robustness of a routine and is an important measure for translation of a diagnostic tool into clinical practice. For applying deep learning to single‐cell phenotype classification, we are building a computational library *Deepometry*, available at https://github.com/broadinstitute/deepometry.

In addition, in order to improve the machine‐learning model performance, model selection could be applied to optimize the parameters. In this work, different machine‐learning methods as well as different preprocessing approaches have been compared, but no model selection has been applied; the default settings and parameters from the scikit‐learn packages were kept. In model selection, for each machine‐learning method, various parameter combinations would be tested to determine the one that leads to the best model performance.

Another open question is to apply unsupervised learning, and/or other methods of dimension reduction and visualization, for example, principal component analysis (PCA), or uniform manifold approximation and projection 17 and to interpret the patterns of cell subpopulations and their phenotypic similarity. This could potentially define new WBC subpopulations and gain insights into their role when combined with qualitative and quantitative cluster analysis (e.g.,, differentiate neutrophil clusters in asthma patients vs. controls). In addition, unsupervised machine learning can be applied to further investigate the label‐free classification potential of lymphocytes into B and T cells by analyzing the resulting morphological characteristics in the B and T cell clusters to improve the supervised machine‐learning model and give a better explanation of the morphology‐based characterization of lymphocytes, which is subject to further research.

Before the methods developed here can be applied in the clinic, certain limitations in the presented work must be addressed. First, the presented machine‐learning models would only be expected to achieve high accuracy on samples using the same instrument and sample preparation techniques. For use in other settings, a new model would need to be trained specifically for each laboratory's setup; we have provided here procedures and software to do so. Alternately, a more robust and trustworthy universal model could be created and validated on a larger amount of labeled data from a variety of laboratories and instruments, an approach that was recently successful for a nucleus detection model trained across a huge variety of cell types and microscopes in the 2018 Data Science Bowl (https://www.kaggle.com/c/data-science-bowl-2018). Such an effort would also allow confirming the accuracy of the best algorithm identified in this study, the “winner” would be expected to show optimistic accuracy rates. Finally, for adoption of these methods into the clinic, it would be key for clinicians to determine what are the right sensitivity and specificity metrics for a given test, what level of accuracy is sufficient for clinical use, and what user interface is most effective for generating and interpreting the results. Our study provides the first evidence that the pursuit of this goal will likely be worthwhile.

Addressing these limitations could lead to the application of the proposed label‐free WBC classification method in the clinic, saving the time, and cost of immunofluorescence labeling of WBCs and obviating many of the current challenges in clinical immunology. For example, clinical immune monitoring studies can be confounded by factors such as differences in blood sample preparation that can affect antibody binding, antibody clonal variability, differences in fluorochrome stability, diversity in flow cytometer lasers and detectors from lab to lab, and subjective population gating and analysis 18. The method described here, if proven sufficiently accurate and reproducible, could lead to significant improvements in data quality and reliability for longitudinal studies. Finally, the ability to characterize live cells in the absence of fluorescence staining leaves open the possibility of collecting the cells for downstream functional analyses or even re‐introduction of subpopulations to the patient.

## Conflict of Interest

D.K.F. is employed by Luminex Corporation, the maker of the Amnis ImageStream instrument used in this study. D.K.F. is also founder of Autograph Biosciences, Inc. No other potential conflicts of interest were reported by the authors.

## Supporting information


**Appendix S1:** Supplementary Information

**Appendix S2:** Supporting Information
