# Supplementary material for: Label‐Free Identification of White Blood Cells Using Machine Learning
Source: Cytometry A. 2019 May 13;95(8):836–42. doi: 10.1002/cyto.a.23794 (PMC6767740; doi:10.1002/cyto.a.23794)
Supplement: Supplementary file 1 — Appendix S1: Supplementary Information [file CYTO-95-836-s001.docx]

### Supplementary Material

#### (A) Code availability

**Code A.1: Script for subject-wise cross-validation machine learning.**

| from __future__ import division from sklearn.naive_bayes import GaussianNB from sklearn.ensemble import RandomForestClassifier from sklearn.ensemble import GradientBoostingClassifier from sklearn.svm import SVC from sklearn.neighbors import KNeighborsClassifier from sklearn.ensemble import AdaBoostClassifier from sklearn.metrics import confusion_matrix from sklearn import preprocessing from sklearn.feature_selection import VarianceThreshold from imblearn.over_sampling import SMOTE import fnmatch import numpy import operator import os import pandas import sklearn import sys import utils   def loso_cv_main(classification_type, balanced, preprocessing, threshold, names_classifiers):  if classification_type == 'all':  cell_types = ['lymphocyte', 'eosinophil', 'monocyte', 'neutrophil']  elif classification_type == 'lymphocytes':  cell_types = ['B', 'T']   input_dir = ''  main_output_dir = ''    if preprocessing == '':  preprocessing_ = 'no_preprocessing'  else:  preprocessing_ = preprocessing  if preprocessing == 'low_var':  output_dir = main_output_dir + classification_type.capitalize() + '/' + balanced + '/' + preprocessing_ + '_' + str(threshold)  else:  output_dir = main_output_dir + classification_type.capitalize() + '/' + balanced + '/' + preprocessing_  if not os.path.exists(output_dir):  os.makedirs(output_dir)   #Get the intersection of the zero score features over all subjects  if preprocessing == 'remove_zero_features':  if classification_type == 'all':  zero_features_list_path = main_output_dir + 'All/' + balanced + '/no_preprocessing'  elif classification_type == 'lymphocytes':  zero_features_list_path = main_output_dir + 'Lymphocytes/' + balanced + '/no_preprocessing'  files = fnmatch.filter(os.listdir(zero_features_list_path), 'GradientBoosting_feature_importances_*.txt')  zero_features_list = []  os.chdir(zero_features_list_path)  for filename in files:  lines = [line.rstrip('\n') for line in open(filename)]  del lines[0]  l = [e.split(":") for e in lines]  dic = [[e[0], float(e[1])] for e in l]  dic = dict(dic)  zero_features_list.append([k for k,v in dic.items() if v==0])  zero_features = set(zero_features_list[0]).intersection(*zero_features_list[:1])  utils.write_zero_features_to_file(zero_features, output_dir)   #Get the intersection of the top 10 features over all subjects  if preprocessing == 'top_10_features':  if classification_type == 'all':  top_10_features_path = main_output_dir + 'All/' + balanced + '/no_preprocessing'  elif classification_type == 'lymphocytes':  top_10_features_path = main_output_dir + 'Lymphocytes/' + balanced + '/no_preprocessing'  files = fnmatch.filter(os.listdir(top_10_features_path), 'GradientBoosting_feature_importances_*.txt')  best_dicts_list = []  os.chdir(top_10_features_path)  for filename in files:  lines = [line.rstrip('\n') for line in open(filename)]  del lines[0]  l = [e.split(":") for e in lines]  dic = [[e[0], float(e[1])] for e in l]  dic = dict(dic)  best_dicts_list.append({k: v for k,v in dic.items() if v>=0.01})  best_features_list = [set(dic.keys()) for dic in best_dicts_list]  best_features_set = set.intersection(*best_features_list)  best_scores = []  best_dict = dict()  for feature in best_features_set:  best_scores = [dic.get(feature) for dic in best_dicts_list]  best_dict.update({feature: numpy.mean(numpy.asarray(best_scores))})  sorted_dict = sorted(best_dict.items(), key=operator.itemgetter(1))  sorted_dict.reverse()  sorted_dict = dict(sorted_dict)  top_10_features = list(sorted_dict.keys())  utils.write_top_10_features_to_file(sorted_dict, output_dir)   #Features to exclude  exclude_featuresDF = ['Location_CenterMassIntensity_X_DF_image', 'Location_CenterMassIntensity_Y_DF_image', 'Location_Center_X', 'Location_Center_Y', 'Location_MaxIntensity_X_DF_image', 'Location_MaxIntensity_Y_DF_image', 'Number_Object_Number']  exclude_featuresBF = ['ImageNumber', 'ObjectNumber', 'AreaShape_Center_X', 'AreaShape_Center_Y', 'AreaShape_EulerNumber', 'AreaShape_Orientation', 'AreaShape_Solidity', 'Location_CenterMassIntensity_X_BF_image', 'Location_CenterMassIntensity_Y_BF_image', 'Location_Center_X', 'Location_Center_Y', 'Location_MaxIntensity_X_BF_image', 'Location_MaxIntensity_Y_BF_image', 'Number_Object_Number']  exclude_featuresDF.extend(exclude_featuresBF)  exclude_features = exclude_featuresDF   subject_ids = next(os.walk(input_dir))[1]   data_filename = 'BF_cells_on_grid.txt';    num_cells = [] #num_cells =[p1_num_cells, p2_num_cells, ..]: pi_num_cells = [#label1, #label2, ..]  data = []  for subject_id in subject_ids:  subject_data = []  num_cells_subject = []  for cell_type in cell_types:  if cell_type == 'lymphocyte':  subject_cell_type_data = []  for sub_label in ['B', 'T']:  subject_sub_label_data = pandas.read_csv("".join([input_dir, "/", subject_id, "/", sub_label, "/", data_filename]), sep='\t')  subject_cell_type_data.append(subject_sub_label_data)  subject_cell_type_data = pandas.concat(subject_cell_type_data)  else:  subject_cell_type_data = pandas.read_csv("".join([input_dir, "/", subject_id, "/", cell_type, "/", data_filename]), sep='\t')  ground_truth_list = [cell_type] * len(subject_cell_type_data)  subject_cell_type_data = subject_cell_type_data.drop(exclude_features, axis=1)  subject_cell_type_data = subject_cell_type_data[subject_cell_type_data.columns.drop(list(subject_cell_type_data.filter(regex='Marker_image')))]  if preprocessing == 'remove_zero_features':  subject_cell_type_data = subject_cell_type_data[subject_cell_type_data.columns.drop(zero_features)]  if preprocessing == 'top_10_features':  subject_cell_type_data = subject_cell_type_data[top_10_features]  subject_cell_type_data = subject_cell_type_data.assign(ground_truth = ground_truth_list)  subject_cell_type_data = subject_cell_type_data.dropna()  subject_data.append(subject_cell_type_data)  num_cells_subject.append(subject_cell_type_data.shape[0])  if balanced == 'imbalanced':  subject_data = pandas.concat(subject_data)  subject_data = subject_data.dropna()  data.append(subject_data)  num_cells.append(num_cells_subject)    #Get equal nums of cells from each cell type (balanced classes) Random Undersampling  if balanced == 'undersampled':  for j in range(0, len(data)):  min_num_cells = min(num_cells[j])  for i in range(0, len(cell_types)):  data[j][i] = data[j][i].sample(min_num_cells)  data[j] = pandas.concat(data[j])   #Separate ground truth from the rest  subjects_ground_truth = [subject_data['ground_truth'] for subject_data in data] #subjects_ground_truth = [subject1_ground_truth, subject2_ground_truth,..]  data = [subject_data.drop('ground_truth', axis = 1) for subject_data in data]   utils.write_num_cells_to_file(num_cells, subject_ids, cell_types, output_dir)   #Leave one subject out cross validation  for name, classifier in names_classifiers:  for i in range(0,len(data)):  #combine pandas train frames  train_data = pandas.concat(data[:i]+data[i+1:])  #Features standardization using z-score normalization  if preprocessing == 'norm':  norm_train_data = pandas.DataFrame(columns=train_data.columns)  for feature_name in train_data.columns:  norm_train_data[feature_name] = (train_data[feature_name] - train_data[feature_name].mean()) / train_data[feature_name].std()  train_data = norm_train_data  #Remove low variance features  if preprocessing == 'low_var':  selector = VarianceThreshold(pow(10, threshold))  columns = train_data.columns  train_data = selector.fit_transform(train_data)  labels = [columns[i] for i in selector.get_support(indices=True)]  train_data = pandas.DataFrame(train_data, columns=labels)  train_ground_truth = pandas.concat(subjects_ground_truth[:i]+subjects_ground_truth[i+1:])  test_data = data[i]  #Remove the features with low variance (also removed from the train data) from the test data  if preprocessing == 'low_var':  test_data = test_data[labels]  #Features standardization of the test data  #This is done separately to avoid the flow of info from train to test data  if preprocessing == 'norm':  norm_test_data = pandas.DataFrame(columns=test_data.columns)  for feature_name in test_data.columns:  norm_test_data[feature_name] = (test_data[feature_name] - test_data[feature_name].mean()) / test_data[feature_name].std()  test_data = norm_test_data  test_ground_truth = subjects_ground_truth[i]  #Train the classifier  classifier.fit(train_data, train_ground_truth)  #Write feature importances to file for each subject  if name in ['RandomForest', 'GradientBoosting', 'AdaBoost'] :  feature_names = train_data.columns  feature_importances = sorted(zip(classifier.feature_importances_, feature_names),reverse=True)  utils.write_feature_importances_to_file(feature_importances, i, name, output_dir)  #Classify the test data using the trained classifier  prediction = classifier.predict(test_data)  #Compute the confusion matrix  cm = confusion_matrix(test_ground_truth, prediction, labels = cell_types)  #Write each confusion matrix to file  utils.write_ith_cm_to_file(name, i ,cm, output_dir) |
| --- |

**Code A.2: A function to iterate subject-wise cross validation over several conditions.**

| from sklearn.naive_bayes import GaussianNB from sklearn.ensemble import RandomForestClassifier from sklearn.ensemble import GradientBoostingClassifier from sklearn.svm import SVC from sklearn.neighbors import KNeighborsClassifier from sklearn.ensemble import AdaBoostClassifier from loso_cv_main import loso_cv_main   names_classifiers = [] names_classifiers.append(('NaiveBayes', GaussianNB())) names_classifiers.append(('RandomForest', RandomForestClassifier())) names_classifiers.append(('GradientBoosting', GradientBoostingClassifier())) names_classifiers.append(('KNN', KNeighborsClassifier())) names_classifiers.append(('SVC', SVC())) names_classifiers.append(('AdaBoost', AdaBoostClassifier()))   preprocessing_types = ['', 'low_var', 'norm', 'remove_zero_features', 'top_10_features'] thresholds = [-3, -4, -5] for classification_type in ['lymphocytes', 'all']:  #for balanced in ['undersampled', 'oversampled', 'imbalanced']:  for balanced in ['imbalanced', 'balanced']:  for preprocessing in preprocessing_types:  if preprocessing == 'low_var':  for threshold in thresholds:  loso_cv_main(classification_type, balanced, preprocessing, threshold, names_classifiers)  else:  loso_cv_main(classification_type, balanced, preprocessing, None, names_classifiers) |
| --- |

**Code A.3: utils.py helper functions.**

| import os  def write_num_cells_to_file(num_cells, subject_ids, cell_types, output_dir):  os.chdir(output_dir)  num_cells_file = open('number_of_cels.txt', 'w+')  for i in range(0, len(num_cells)):  num_cells_file.write(subject_ids[i]+ " ")  for j in range(0,len(cell_types)):  num_cells_file.write(" " + cell_types[j]+ ": " + str(num_cells[i][j]))  num_cells_file.write("\n")  num_cells_file.close()     def write_top_10_features_to_file(top_10_features_dic, output_dir):  os.chdir(output_dir)  top_10_features_file = open('used_top_10_features.txt', 'w+')  for feature, score in top_10_features_dic.items():  top_10_features_file.write(feature + ': ' + str(score) + '\n')  top_10_features_file.close()     def write_zero_features_to_file(zero_features, output_dir):  os.chdir(output_dir)  zero_features_file = open('zero_features.txt', 'w+')  for feature in zero_features:  zero_features_file.write(feature + '\n')  zero_features_file.close() |
| --- |

**Code A.4: stitching.py script to create montages from image data contained in .CIF files.**

| #!/bin/bash  populations_path="" montages_path="" stitching_script_path=""  for patient_path in $populations_path/*; do  patient_id=$(basename $patient_path)  echo $patient_id  mkdir $montages_path/$patient_id  for population_path in $patient_path/*; do  echo $population_path  cell_type_name=$(basename $population_path)  mkdir $montages_path/$patient_id/$cell_type_name  i=1  for batch_cif_path in $population_path/*; do  echo $batch_cif_path  output_path=$montages_path/$patient_id/$cell_type_name/"batch"$i  image_path=$batch_cif_path  python3 $stitching_script_path -o $output_path $image_path  rm $output_path/ch2*  rm $output_path/ch4*  rm $output_path/ch6*  i=$(expr $i + 1)  done  done done |
| --- |

#### (B) CellProfiler Pipeline

WBC_ML.cppipe

####

#### (C) Machine learning classifiers comparison results

| F1-score % | Lymphocytes | Eosinophils | Monocytes | Neutrophils |
| --- | --- | --- | --- | --- |
| AdaBoost | 98 | 38 | 21 | 75 |
| GB | 99 | 92 | 83 | 99 |
| KNN | 99 | 82 | 77 | 98 |
| NB | 76 | 60 | 36 | 89 |
| RF | 99 | 90 | 80 | 99 |
| SVM | 99 | 58 | 78 | 98 |
| **Table C.1: Results of classifying imbalanced WBC classes.** The table compares the F1-score results of classifying imbalanced WBC classes for each WBC type for six ML algorithms. | | | | |

|  | GB F1-score % | | | |
| --- | --- | --- | --- | --- |
|  | Lymphocytes | Eosinophils | Monocytes | Neutrophils |
| Run0 | 98 | 97 | 96 | 97 |
| Run1 | 97 | 97 | 96 | 97 |
| Run2 | 98 | 94 | 97 | 95 |
| Run3 | 98 | 95 | 97 | 96 |
| Run4 | 98 | 97 | 96 | 97 |
| Run5 | 98 | 97 | 97 | 96 |
| Run6 | 94 | 97 | 95 | 97 |
| Run7 | 96 | 97 | 95 | 97 |
| Run8 | 98 | 96 | 97 | 96 |
| Run9 | 94 | 96 | 94 | 96 |
| **Table C.2: Random undersampling results from ten WBC classification tests.** The table shows the results of classifying random undersampled WBC classes using Gradient Boosting for ten runs. | | | | |

| F1-score % | B-Lymphocytes | T-Lymphocytes |
| --- | --- | --- |
| AdaBoost | 49 | 92 |
| GB | 50 | 93 |
| KNN | 33 | 90 |
| NB | 46 | 61 |
| RF | 48 | 91 |
| SVM | 35 | 93 |
| **Table C.1: Results of classifying imbalanced lymphocyte classes.** The table compares the F1-score results of classifying imbalanced lymphocyte classes for six ML algorithms. | | |

|  | GB F1-score % | |
| --- | --- | --- |
|  | B-Lymphocytes | T-Lymphocytes |
| Run0 | 79 | 76 |
| Run1 | 79 | 75 |
| Run2 | 80 | 76 |
| Run3 | 79 | 76 |
| Run4 | 79 | 75 |
| Run5 | 80 | 76 |
| Run6 | 80 | 76 |
| Run7 | 79 | 76 |
| Run8 | 79 | 75 |
| Run9 | 80 | 76 |
| **Table C.2: Random undersampling results from ten lymphocyte classification tests.** The table shows the results of classifying random undersampled lymphocyte classes using Gradient Boosting for ten runs. | | |

#### (D) Subject-wise cross validation


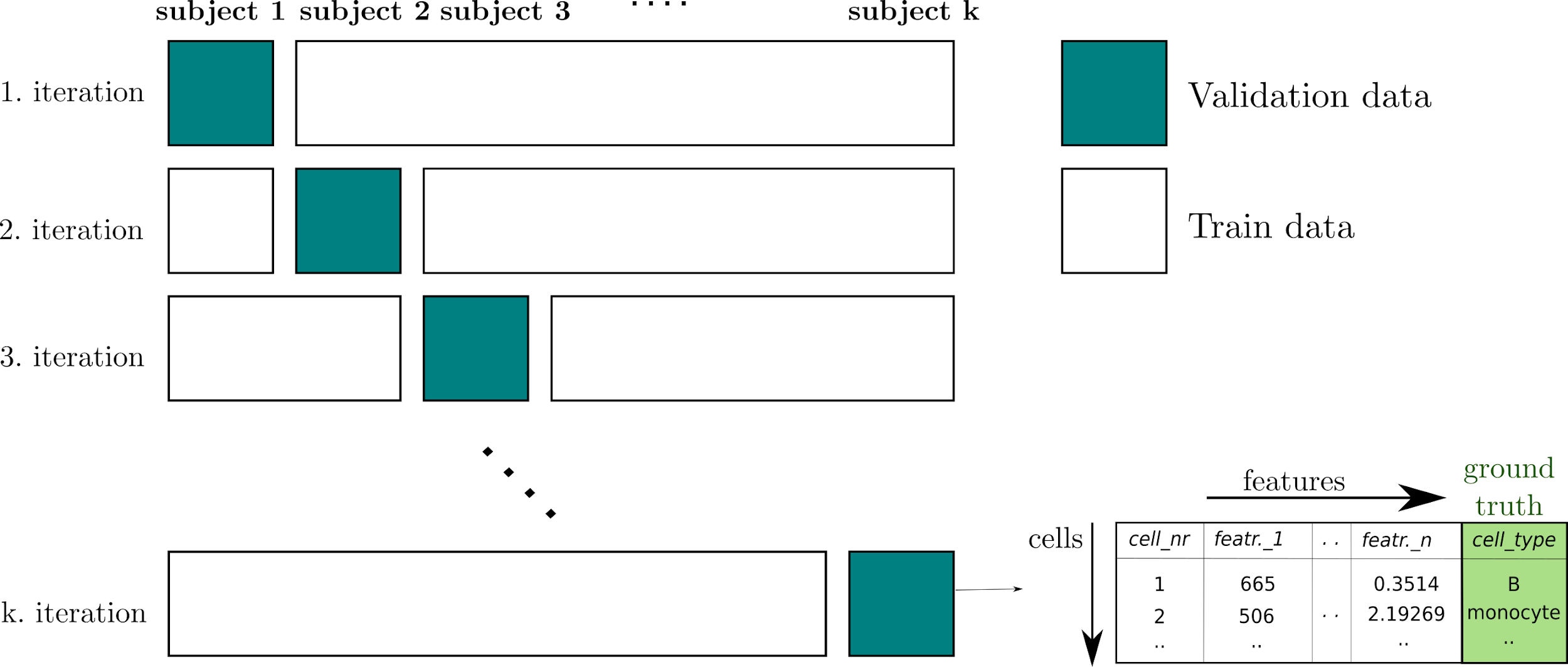


**Figure D.1: Subject-wise cross validation.** In each iteration over the datasets, one subject was separated from the data pool and used for validation; the remaining data was pooled and used for training the machine learning classifier.

#### (E) Ratio of cells in-focus and single cells

| Subject | Sample | Cells in-focus | Single cells |
| --- | --- | --- | --- |
| CRF049 | unstained | 95 % | 47,8 % |
|  | CD3 | 88,5 % | 53,9 % |
|  | CD14 | 92,7 % | 61,6 % |
|  | CD15 | 76,5 % | 43,1% |
|  | CD19 | 94 % | 58,3 % |
| CRF101 | unstained | 97 % | 56,3% |
|  | CD3 | 92,3 % | 53 % |
|  | CD14 | 96,8 % | 50,2 % |
|  | CD15 | 93,8 % | 47,5 % |
|  | CD19 | 93 % | 42,5 % |
| CRF102 | unstained | 97 % | 71,5 % |
|  | CD3 | 95 % | 73,5 % |
|  | CD14 | 97 % | 74,1 % |
|  | CD15 | 95,2 % | 67,9 % |
|  | CD19 | 96,7 % | 70,2 % |
| CRF130 | unstained | 95 % | 44,2 % |
|  | CD3 | 94,3 % | 44,3 % |
|  | CD14 | 94,1 % | 39,4 % |
|  | CD15 | 86,4 % | 28,4 % |
|  | CD19 | 93,6 % | 34,2 % |
| CRF59 | unstained | 95,8 % | 43,2 % |
|  | CD3 | 92 % | 27,6 % |
|  | CD14 | 79,7 % | 46,1 % |
|  | CD15 | 77 % | 12,9 % |
|  | CD19 | 93,2 % | 41,5 % |
| CRF022 | unstained | 96,2 % | 70,5 % |
|  | CD3 | 95,8 % | 72 % |
|  | CD14 | 98 % | 74,7 % |
|  | CD15 | 85,2 % | 55 % |
|  | CD19 | 94,3 % | 55,8 % |
| CRF034 | unstained | 97,7 % | 37,7 % |
|  | CD3 | 96,7 % | 21,2 % |
|  | CD14 | 99 % | 37,8 % |
|  | CD15 | 86 % | 15,2 % |
|  | CD19 | 98,2 % | 28,1 % |
| CRF041 | unstained | 92 % | 70,8 % |
|  | CD3 | 85,9 % | 61,9 % |
|  | CD14 | 92,8 % | 74 % |
|  | CD15 | 56,3 % | 31 % |
|  | CD19 | 94,4 % | 74,8 % |
| CRF066 | unstained | 97,7 % | 37,7 % |
|  | CD3 | 96,7 % | 21,2 % |
|  | CD14 | 99 % | 37,8 % |
|  | CD15 | 94,3 % | 27,5 % |
|  | CD19 | 98 % | 42,5 % |
| CRF074 | unstained | 98,9 % | 47,4 % |
|  | CD3 | 98,1 % | 30 % |
|  | CD14 | 97,6 % | 22,3 % |
|  | CD15 | 92,6 % | 19,6 % |
|  | CD19 | 98,8 % | 30,8 % |
| CRF101_2015 | unstained | 98,1 % | 53,3 % |
|  | CD3 | 98,2 % | 33,6 % |
|  | CD14 | 98,4 % | 26,7 % |
|  | CD15 | 97,6 % | 23,7 % |
|  | CD19 | 98,1 % | 22,8 % |
| CRF132 | unstained | 92,7 % | 54,5 % |
|  | CD3 | 92,5 % | 61,1 % |
|  | CD14 | 96,3 % | 65,8 % |
|  | CD15 | 95,4 % | 54,5 % |
|  | CD19 | 94,7 % | 75,2 % |
| CRF186 | unstained | 94,2 % | 65,3 % |
|  | CD3 | 95,3 % | 70,9 % |
|  | CD14 | 97,2 % | 74,7 % |
|  | CD15 | 87,1 % | 58,8 % |
|  | CD19 | 96,2 % | 75,9 % |

**Table E.1: Ratio of in-focus and single cells to the total number of cells.** The table contains the percents of in-focus and single cells to the total number of cells for each sample of each blood donor. The in-focus percentages were subjective to the cutoff of what is considered 'in-focus'. The relatively low percentage of single cells in some cases was due to the high amount of debris.

#### (F) Number of White Blood Cells used for cross-validation

| Subject | B-cells | T-cells | Eosinophils | Monocytes | Neutrophils |
| --- | --- | --- | --- | --- | --- |
| CRF022 | 388 | 1806 | 271 | 268 | 1601 |
| CRF034 | 126 | 1106 | 287 | 176 | 1386 |
| CRF041 | 146 | 1822 | 121 | 115 | 3172 |
| CRF049 | 167 | 1372 | 603 | 292 | 4030 |
| CRF59 | 130 | 753 | 112 | 3 | 1287 |
| CRF066 | 458 | 1704 | 309 | 173 | 4001 |
| CRF074 | 75 | 777 | 196 | 233 | 3780 |
| CRF101_14 | 96 | 579 | 252 | 32 | 7397 |
| CRF101_15 | 84 | 696 | 188 | 190 | 3787 |
| CRF102 | 306 | 2057 | 337 | 509 | 8496 |
| CRF130 | 276 | 1956 | 380 | 376 | 2066 |
| CRF132 | 544 | 4478 | 150 | 615 | 5621 |
| CRF186 | 357 | 1264 | 835 | 432 | 10083 |

**Table F.1: Number of White Blood Cells of each type used for cross-validation.** Each row of the table contains the number of white blood cells of each type for one blood donor.

#### (G) List of all features extracted by CellProfiler

- Intensity_MADIntensity_DF_image
- Intensity_StdIntensity_DF_image
- Intensity_IntegratedIntensity_DF_image
- Intensity_LowerQuartileIntensity_BF_image
- Intensity_UpperQuartileIntensity_DF_image
- Granularity_1_DF_image
- Granularity_1_BF_image
- Intensity_IntegratedIntensityEdge_DF_image
- Intensity_StdIntensityEdge_BF_image
- Intensity_MeanIntensity_BF_image
- Intensity_MeanIntensity_DF_image
- Intensity_MeanIntensityEdge_BF_image
- Intensity_StdIntensity_BF_image
- RadialDistribution_MeanFrac_DF_image_4of4
- RadialDistribution_MeanFrac_BF_image_3of4
- AreaShape_MajorAxisLength
- Intensity_IntegratedIntensityEdge_BF_image
- Texture_AngularSecondMoment_BF_image_3_0
- Intensity_UpperQuartileIntensity_BF_image
- RadialDistribution_FracAtD_DF_image_4of4
- RadialDistribution_MeanFrac_BF_image_4of4
- RadialDistribution_FracAtD_BF_image_2of4
- AreaShape_Eccentricity
- RadialDistribution_MeanFrac_BF_image_2of4
- Intensity_MeanIntensityEdge_DF_image
- AreaShape_MinorAxisLength
- Texture_Variance_BF_image_3_90
- Texture_AngularSecondMoment_BF_image_3_45
- AreaShape_Zernike_0_0
- Texture_AngularSecondMoment_DF_image_3_0
- AreaShape_Zernike_4_0
- Texture_SumAverage_BF_image_3_90
- Intensity_MADIntensity_BF_image
- AreaShape_Compactness
- RadialDistribution_RadialCV_BF_image_1of4
- RadialDistribution_FracAtD_DF_image_2of4
- RadialDistribution_RadialCV_BF_image_3of4
- Intensity_MinIntensityEdge_BF_image
- RadialDistribution_RadialCV_DF_image_4of4
- Intensity_MedianIntensity_DF_image
- Intensity_MedianIntensity_BF_image
- AreaShape_Zernike_7_7
- Granularity_2_BF_image
- RadialDistribution_RadialCV_BF_image_4of4
- Intensity_IntegratedIntensity_BF_image
- Texture_SumAverage_BF_image_3_0
- Intensity_MaxIntensityEdge_BF_image
- RadialDistribution_RadialCV_BF_image_2of4
- AreaShape_Zernike_5_5
- Intensity_MassDisplacement_DF_image
- Intensity_LowerQuartileIntensity_DF_image
- Texture_Correlation_BF_image_3_90
- Intensity_MaxIntensity_DF_image
- Texture_DifferenceEntropy_DF_image_3_0
- Texture_Gabor_DF_image_3
- AreaShape_Zernike_9_5
- AreaShape_Extent
- Granularity_4_DF_image
- RadialDistribution_MeanFrac_DF_image_2of4
- Texture_AngularSecondMoment_BF_image_3_90
- RadialDistribution_RadialCV_DF_image_2of4
- Texture_AngularSecondMoment_BF_image_3_135
- Texture_Variance_BF_image_3_0
- RadialDistribution_FracAtD_DF_image_3of4
- Granularity_5_BF_image
- RadialDistribution_MeanFrac_DF_image_3of4
- Granularity_3_DF_image
- Intensity_MaxIntensity_BF_image
- RadialDistribution_FracAtD_BF_image_1of4
- AreaShape_Zernike_1_1
- AreaShape_Zernike_8_6
- AreaShape_Zernike_3_3
- RadialDistribution_FracAtD_DF_image_1of4
- RadialDistribution_FracAtD_BF_image_3of4
- AreaShape_MaxFeretDiameter
- AreaShape_Zernike_9_7
- AreaShape_Zernike_6_0
- Intensity_MassDisplacement_BF_image
- Texture_SumEntropy_BF_image_3_90
- RadialDistribution_MeanFrac_DF_image_1of4
- RadialDistribution_RadialCV_DF_image_3of4
- AreaShape_MinFeretDiameter
- Texture_InfoMeas2_DF_image_3_135
- Texture_SumVariance_BF_image_3_45
- Granularity_5_DF_image
- AreaShape_Zernike_5_3
- AreaShape_MeanRadius
- Texture_Variance_DF_image_3_45
- Texture_InverseDifferenceMoment_BF_image_3_0
- Granularity_2_DF_image
- Intensity_MinIntensity_BF_image
- Texture_AngularSecondMoment_DF_image_3_45
- Texture_SumAverage_DF_image_3_0
- AreaShape_Zernike_9_9
- Intensity_MaxIntensityEdge_DF_image
- Texture_Variance_DF_image_3_0
- Texture_SumEntropy_BF_image_3_0
- Texture_Gabor_BF_image_3
- AreaShape_Zernike_8_8
- Texture_InverseDifferenceMoment_BF_image_3_135
- Granularity_4_BF_image
- AreaShape_Zernike_6_4
- Texture_DifferenceVariance_DF_image_3_135
- Texture_DifferenceEntropy_BF_image_3_0
- Texture_Correlation_BF_image_3_0
- Texture_Contrast_DF_image_3_135
- Texture_InfoMeas2_BF_image_3_45
- Texture_InfoMeas2_DF_image_3_90
- Texture_DifferenceVariance_BF_image_3_135
- Texture_InfoMeas1_BF_image_3_45
- Texture_Entropy_BF_image_3_0
- Texture_Entropy_DF_image_3_90
- AreaShape_Zernike_7_1
- Texture_SumEntropy_BF_image_3_135
- Texture_InfoMeas1_DF_image_3_0
- AreaShape_Zernike_7_5
- AreaShape_Zernike_8_0
- AreaShape_FormFactor
- Texture_Correlation_DF_image_3_0
- Texture_Contrast_DF_image_3_45
- Texture_Entropy_BF_image_3_135
- AreaShape_Zernike_4_2
- Intensity_MinIntensityEdge_DF_image
- Texture_AngularSecondMoment_DF_image_3_90
- Texture_SumVariance_BF_image_3_0
- Texture_Correlation_DF_image_3_135
- RadialDistribution_RadialCV_DF_image_1of4
- Texture_DifferenceVariance_BF_image_3_90
- Texture_InfoMeas2_BF_image_3_90
- Texture_InfoMeas1_BF_image_3_135
- Texture_InfoMeas1_DF_image_3_135
- Texture_Variance_DF_image_3_135
- AreaShape_Area
- RadialDistribution_FracAtD_BF_image_4of4
- AreaShape_Zernike_9_1
- Texture_Variance_BF_image_3_45
- Texture_Contrast_BF_image_3_135
- Texture_DifferenceVariance_DF_image_3_45
- Texture_Entropy_BF_image_3_45
- AreaShape_Zernike_8_4
- AreaShape_Zernike_6_2
- Texture_Contrast_DF_image_3_90
- AreaShape_Zernike_5_1
- Intensity_MinIntensity_DF_image
- AreaShape_Zernike_4_4
- Texture_DifferenceVariance_DF_image_3_90
- Texture_SumEntropy_DF_image_3_135
- Intensity_StdIntensityEdge_DF_image
- AreaShape_Zernike_7_3
- Texture_DifferenceVariance_BF_image_3_45
- Texture_SumVariance_BF_image_3_90
- Texture_Correlation_BF_image_3_135
- Texture_Entropy_DF_image_3_0
- Texture_SumVariance_DF_image_3_0
- Texture_InfoMeas2_BF_image_3_135
- Texture_Contrast_DF_image_3_0
- Texture_SumEntropy_DF_image_3_45
- Texture_AngularSecondMoment_DF_image_3_135
- Texture_InfoMeas1_DF_image_3_90
- Texture_InfoMeas1_BF_image_3_90
- Texture_InfoMeas2_DF_image_3_45
- Texture_InverseDifferenceMoment_BF_image_3_45
- Texture_SumEntropy_DF_image_3_90
- Texture_Correlation_BF_image_3_45
- Texture_InfoMeas2_BF_image_3_0
- Granularity_3_BF_image
- Texture_Entropy_DF_image_3_135
- Texture_InverseDifferenceMoment_DF_image_3_90
- AreaShape_Zernike_9_3
- Texture_InfoMeas1_BF_image_3_0
- Texture_SumAverage_DF_image_3_45
- AreaShape_Zernike_3_1
- Texture_SumAverage_DF_image_3_135
- Texture_DifferenceEntropy_BF_image_3_135
- Texture_SumVariance_BF_image_3_135
- Texture_DifferenceVariance_DF_image_3_0
- Texture_Contrast_BF_image_3_90
- Texture_SumAverage_DF_image_3_90
- Texture_DifferenceEntropy_DF_image_3_45
- Texture_SumAverage_BF_image_3_45
- Texture_DifferenceEntropy_DF_image_3_135
- Texture_SumEntropy_BF_image_3_45
- Texture_Entropy_DF_image_3_45
- RadialDistribution_MeanFrac_BF_image_1of4
- AreaShape_MedianRadius
- Texture_InverseDifferenceMoment_BF_image_3_90
- Texture_InverseDifferenceMoment_DF_image_3_135
- AreaShape_Zernike_2_2
- AreaShape_Perimeter
- Texture_DifferenceEntropy_BF_image_3_90
- Texture_SumAverage_BF_image_3_135
- Texture_DifferenceVariance_BF_image_3_0
- Texture_InfoMeas2_DF_image_3_0
- Texture_InverseDifferenceMoment_DF_image_3_45
- Texture_Correlation_DF_image_3_45
- Texture_DifferenceEntropy_DF_image_3_90
- Texture_SumVariance_DF_image_3_90
- AreaShape_Zernike_8_2
- Texture_SumVariance_DF_image_3_45
- Texture_Correlation_DF_image_3_90
- Texture_Variance_BF_image_3_135
- Texture_Variance_DF_image_3_90
- Texture_SumVariance_DF_image_3_135
- Texture_SumEntropy_DF_image_3_0
- Texture_InverseDifferenceMoment_DF_image_3_0
- Texture_InfoMeas1_DF_image_3_45
- Texture_Entropy_BF_image_3_90
- Texture_DifferenceEntropy_BF_image_3_45
- Texture_Contrast_BF_image_3_45
- Texture_Contrast_BF_image_3_0
- AreaShape_Zernike_6_6
- AreaShape_Zernike_2_0
- AreaShape_MaximumRadius
